# Anti-senescence ion-delivering nanocarrier for recovering therapeutic properties of long-term-cultured human adipose-derived stem cells

**DOI:** 10.1186/s12951-021-01098-7

**Published:** 2021-10-30

**Authors:** Yeong Hwan Kim, Gwang-Bum Im, Sung-Won Kim, Yu-Jin Kim, Taekyung Yu, Ju-Ro Lee, Soong Ho Um, Yoon Ki Joung, Suk Ho Bhang

**Affiliations:** 1 School of Chemical Engineering, Sungkyunkwan University, Suwon, 440-746 Republic of Korea; 2 Department of Chemical Engineering, College of Engineering, Kyung Hee University, Yongin, 17104 Republic of Korea; 3 Center for Biomaterials, Biomedical Research Institute, Korea Institute of Science and Technology, Hwarang-ro 14-gil 5, Seoungbuk-gu, Seoul, 02792 Republic of Korea; 4 Division of Bio-Medical Science & Technology, University of Science and Technology, 113 Gwahangno, Yuseong-gu, Daejeon, 305-333 Republic of Korea

**Keywords:** Angiogenesis, Functionality restoring, Intracellular ion delivery, Ischemic disease, Senescence, Stem cell therapy

## Abstract

**Background:**

Human adipose-derived stem cells (hADSCs) have been used in various fields of tissue engineering because of their promising therapeutic efficacy. However, the stemness of hADSCs cannot be maintained for long durations, and their therapeutic cellular functions, such as paracrine factor secretion decrease during long-term cell culture. To facilitate the use of long-term-cultured hADSCs (L-ADSCs), we designed a novel therapeutic anti-senescence ion-delivering nanocarrier (AIN) that is capable of recovering the therapeutic properties of L-ADSCs. In the present study, we introduced a low-pH-responsive ion nanocarrier capable of delivering transition metal ions that can enhance angiogenic paracrine factor secretion from L-ADSCs. The AINs were delivered to L-ADSCs in an intracellular manner through endocytosis.

**Results:**

Low pH conditions within the endosomes induced the release of transition metal ions (Fe) into the L-ADSCs that in turn caused a mild elevation in the levels of reactive oxygen species (ROS). This mild elevation in ROS levels induced a downregulation of senescence-related gene expression and an upregulation of stemness-related gene expression. The angiogenic paracrine factor secretion from L-ADSCs was significantly enhanced, and this was evidenced by the observed therapeutic efficacy in response to treatment of a wound-closing mouse model with conditioned medium obtained from AIN-treated L-ADSCs that was similar to that observed in response to treatment with short-term-cultured adipose-derived stem cells.

**Conclusions:**

This study suggests a novel method and strategy for cell-based tissue regeneration that can overcome the limitations of the low stemness and therapeutic efficacy of stem cells that occurs during long-term cell culture.

**Graphical Abstract:**

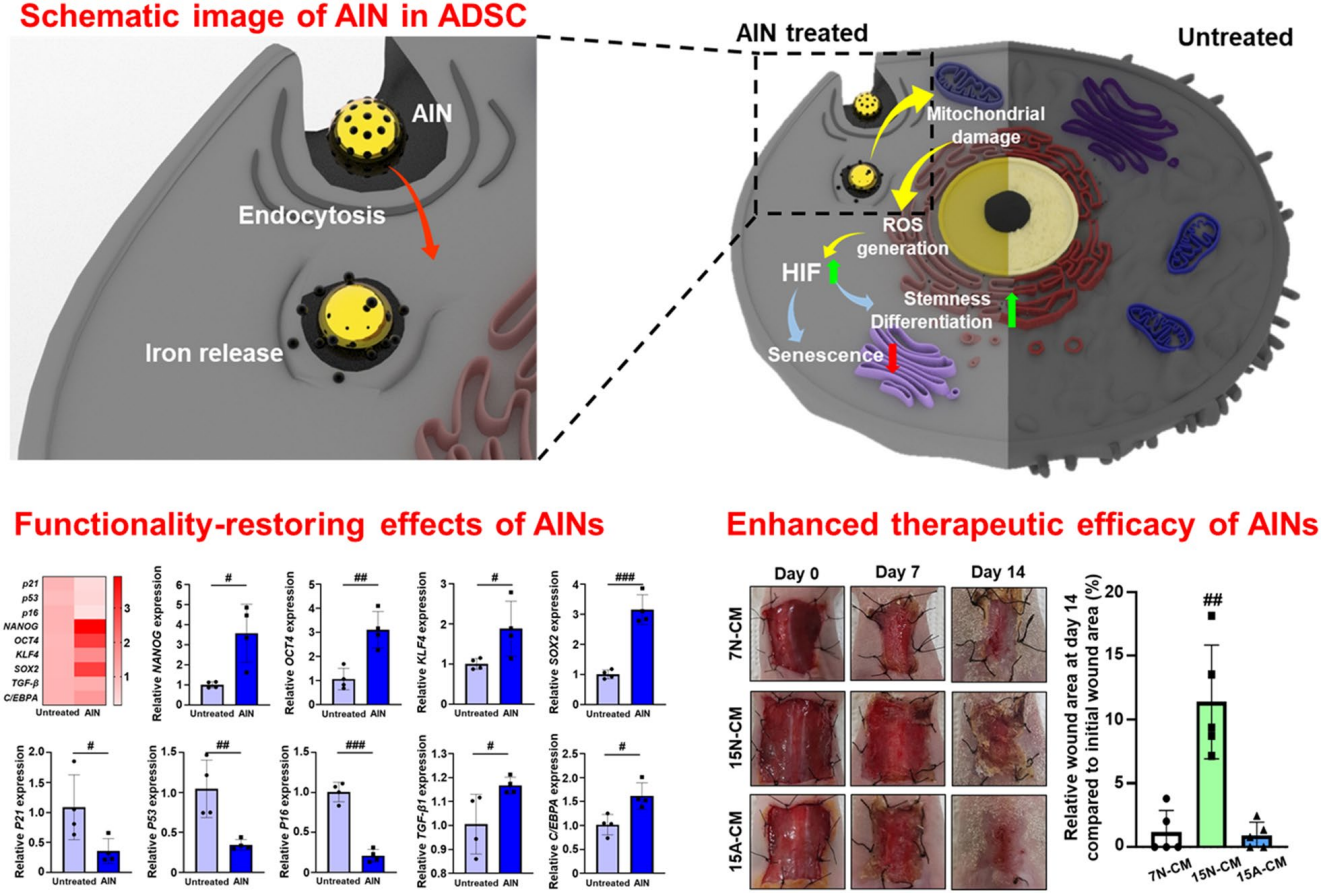

**Supplementary Information:**

The online version contains supplementary material available at 10.1186/s12951-021-01098-7.

## Background

Transition metal-based nanoparticles have been widely studied for use in biomedical applications, such as hyperthermic therapy, drug delivery, and bio-imaging [1, 2]. These nanoparticles have also been applied to stem cells to improve their therapeutic efficacy [3]. Iron (Fe), the representative element of transition metal, has been used to improve the therapeutic effect of stem cell based therapies [4–6]. Despite of its great therapeutic effect, Fe cannot be used in clinical trials due to of its potential toxicity such as ferroptosis. Ferroptosis is a result of iron-dependent oxidative cellular damage, and it can cause neurodegeneration [7, 8]. To utilize the effect of transition metals on the therapeutic efficacy of stem cells without damage, there is a need to accurately control the treatment concentration. In this study, to prohibit the cellular damages such as ferroptosis [9, 10] and enhance the therapeutic effect on stem cells, we first optimized the concentration of Fe for cell treatment and then evaluated the cellular functions.

Stem cell-based therapy has been highlighted as a regenerative medicine approach for vascular diseases [11, 12]. In particular, human adipose-derived stem cells (hADSCs) serve as attractive tools for vascularization and skin regeneration because of their convenient management, ability to differentiate into various cell-types, and secretion of angiogenic factors, such as vascular endothelial growth factor (VEGF), fibroblast growth factor (FGF), and hepatocyte growth factor [13–15]. Despite the therapeutic and differentiation ability of ADSCs, a limited number of clinical trials have been performed using ADSCs because of their short lifespan, quantitative limitations for cell transplantation, and required use at low passage numbers [16, 17]. Cell-free conditioned media (CM) is retrieved from cultured cells and can be used to overcome the aforementioned obstacles. Recently, multiple studies have investigated the validity of the wound-healing effects of cell-free CM [18, 19]. However, long-term-cultured ADSCs (L-ADCSs) secrete low concentrations of angiogenic cytokines, and this makes it difficult to use them as an alternative to short-term-cultured hADSCs (S-ADSCs) in the context of CM application.

Herein, we developed a novel and simple anti-senescence ion-delivering nanocarrier (AIN) and an iron-incorporated gold nanoparticle (AuNP) to enhance the therapeutic efficacy of CM from L-ADSCs. AuNP is well known as non-cytotoxic and stable material and used for bio-imaging material due to their surface plasmon resonance [20]. Only iron ions are released from AINs under the low pH conditions present in late endosomes [21] due to the difference in the reduction potentials of gold and iron [22, 23]. Iron reacts with hydrogen ions and is ionized under the acidic conditions present in late endosomes, while Au remains intact. Iron has been reported to enhance the angiogenic therapeutic efficacy of stem cells by upregulating the expression of the hypoxia-inducible factor (*HIF*) gene that is associated with mitochondrial reactive oxygen species (ROS) [24, 25]. Excessive intracellular ROS levels cause the initiation of programmed cell death [26]; however, mild ROS production induces the upregulation of angiogenic paracrine factor secretion and delays cellular senescence by downregulating senescence-associated gene expression [27–30].

The AINs used in this study can deliver an appropriate quantity of iron ions to hADSCs, thus resulting in no induction of apoptosis in these cells. As a result, iron ions released from the AINs within endosomes generate mild ROS levels, upregulate *HIF* expression, enhance angiogenic therapeutic efficacy, and reverse the senescence of L-ADSCs. This provides meaningful potency to allow for the therapeutic usage of L-ADSCs that until now were considered to be of no use for stem cell therapy. This anti-senescence strategy may expand the biomedical field by allowing for further utilization of stem cell-based therapies.

## Results and discussion

Senescent cells are considered to be bio-waste for stem cell therapy due to their reduced therapeutic efficacy, reduced differentiation ability, and the risk of mutation [31–33]. Strategies for the restoration of the functionality of stem cells, including extracellular modifications and cell reprogramming, have been widely researched [34–37]. However, extracellular modifications are costly and can be time-/labor-consuming and involve complex processes. Also, several studies aimed to overcome the senescence and restore cellular functionality of stem cells using biomaterials have been reported [38–40]. In previous studies, most of biomaterials acted as carriers of genes or drugs. Therefore, additional processes for enhancing gene or drug delivery efficiency were required. Additionally, non-degradable property of biomaterials that can induce materials accumulation in body remained as a problem to be solved. In this study, we used transition metal-based nanoparticle as a therapeutic agent. We designed the nanoparticle to undergo degradation depends on low pH condition. In this study, we developed a safe and simple nanocarrier to restore the functionality of senescent hADSCs. Intracellularly delivered AINs release iron ions in L-ADSCs due to the acidic conditions present in the exosomes, and this release generates mild levels of ROS (Additional file 1: Fig. S1). As a result, the generated mild levels of ROS facilitate a reduction in cellular senescence and restore the functionality of L-ADSCs.

### Characterization of AINs

**Fig. 1:**
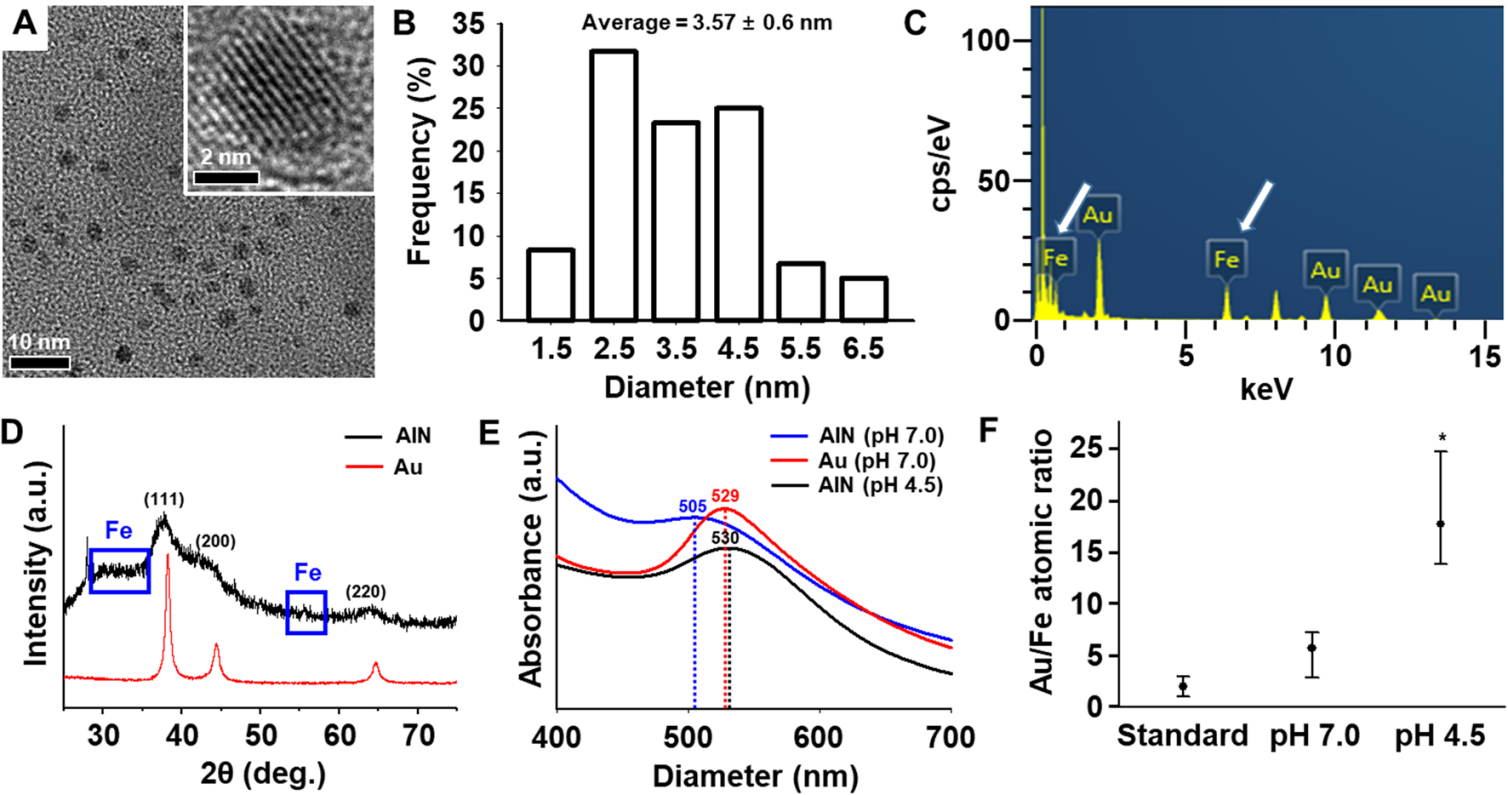
Physical and chemical properties of anti-senescence ion-delivering nanocarriers (AINs).** A** A transmission electron microscopy (TEM) image of AINs (high power magnification at the top-left corner). **B** Size distribution of AINs, as measured using TEM. **C** Energy-dispersive X-ray spectroscopy (EDXS) analysis of AINs. Iron atom peaks are indicated with white arrows. **D** X-ray diffraction (XRD) patterns of AINs. Iron ion patterns are emphasized using blue squares. **E** Ultraviolet-visible spectroscopy (UV-Vis) spectra of AINs at pH 7.0 (blue line), gold nanoparticles (Au) at pH 7.0 (red line), and AINs at pH 4.5 (black line). Peak shifts owing to the present of iron. **F** The component gold/iron ratio profile of AINs as estimated using EDXS under the different pH conditions at 12 h (n = 3, *p < 0.05 versus standard group)

The transmission electron microscopy (TEM) image of the AINs revealed a spherical shape possessing an average diameter of 3.57 ± 0.6 nm (Fig. 1A and B). Energy-dispersive X-ray spectroscopy (EDXS) analysis indicated that the AINs consisted of iron and gold (Fig. 1C). A slight peak movement (blue squares) was detected due to the different sizes of the gold and iron ions based on powder X-ray diffraction (XRD) patterns (Fig. 1D). The ultraviolet/visible (UV-Vis) spectrum peak of the AINs at pH 7.0, was observed at 505 nm according to plasmon resonance, while those of AuNPs and AINs at pH 4.5 were observed at 529 nm and 530 nm according to plasmon resonance, respectively. This demonstrates that the presence of iron causes a red shift (Fig. 1E) [41]. The atomic ratio alteration of AINs under acidic (pH 4.5) and standard (pH 7.0) conditions are presented in Fig. 1F. No significant difference was observed in the atomic ratio (gold/iron) at pH 7.0 compared to that under standard conditions (gold/iron = 2.4), while the same ratio was markedly increased under acidic conditions. The atomic ratio alteration of AINs in response to different pH conditions highlights the potency of AINs in regard to serving as intracellular metal ion carriers to stem cells.

### Optimizing the concentration and treatment time of AINs

**Fig. 2:**
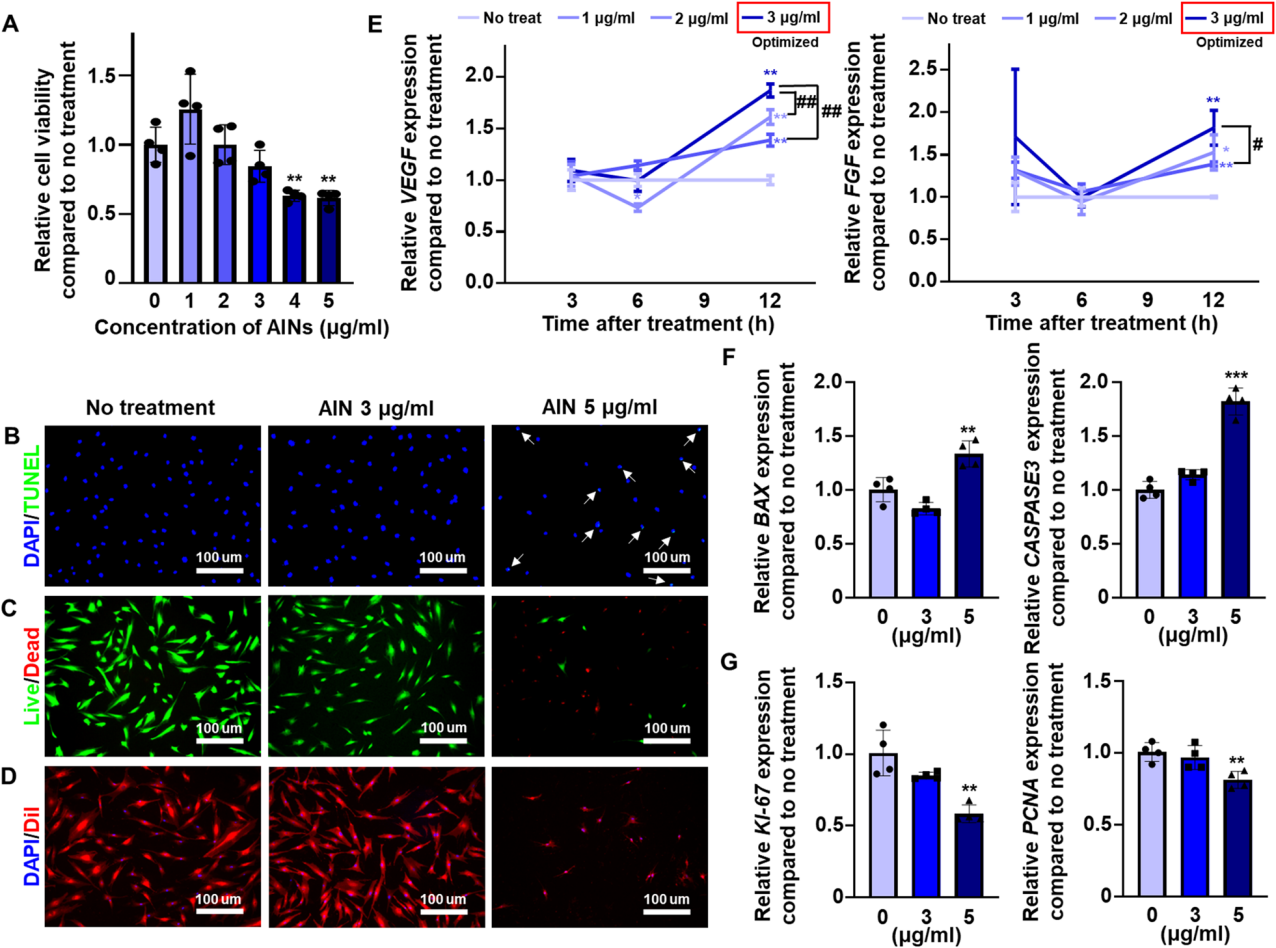
Optimization of the concentration of AINs used for treatment of L-ADSCs.** A** Cell viability of L-ADSCs after treatment with AINs (n = 4, **p < 0.01 versus no treatment group). **B** Terminal deoxynucleotidyl transferase-mediated dUTP nick end labeling (TUNEL) assay examining AIN treated L-ADSCs (blue: nucleus, green: apoptotic cell). White arrows indicate cells undergoing apoptosis that was induced by AINs. Scale bar: 100 μm. **C** Fluorescein diacetate/ethidium (FDA/EB) staining of L-ADSCs post-treatment with AINs (green: live cells, red: dead cells). Scale bar: 100 μm. **D** DiI staining of L-ADSCs treated with AINs (blue: nucleus, red: cellular membrane). Scale bar: 100 μm. **E** Relative mRNA expression levels of *VEGF* and *FGF2* in L-ADSCs at 3, 6, 9, and 12 h after treatment with various concentration of AINs (n = 4, *p < 0.05 and **p < 0.01 versus no treatment group, ^#^*p* < 0.05 and ^##^*p* < 0.01 versus each group). Relative mRNA expression of **F** the apoptosis-related genes *BAX* and *CASPASE3* and **G** the proliferation-related genes *KI-67* and *PCNA* in L-ADSCs after treatment with various concentrations of AINs (n = 3, **p < 0.01 and ***p < 0.001 versus no treatment group)

To optimize the concentration of AINs used for L-ADSC treatment, at cell viability assay was performed in a 24-well cell culture dish using Cell Counting Kit-8 (CCK-8) to verify the cytotoxicity of AINs after treatment for 24 h (Fig. 2 A). AINs exhibited no cytotoxicity up to a concentration of 3 µg/mL of AINs; however, the cytotoxicity was significantly increased at AIN concentrations of greater than 4 µg/mL. Similar to the results of the CCK-8 assay, treatments with 5 µg/mL of AINs induced cell death in L-ADSCs, and at a concentration of 3 µg/mL, AINs exerted no harmful effects on the cell viability of L-ADSCs according to the results of the terminal deoxynucleotidyl transferase-mediated dUTP nick end labeling (TUNEL) assay (Fig. 2B) and the fluorescein diacetate/ethidium bromide (FDA/EB) staining analysis (Fig. 2C). In response to treatment with 5 µg/mL of AINs, L-ADSCs changed morphologically and shrunk in size compared to the characteristics the other groups, thus indicating that a high dose of AINs induced cell apoptosis (Fig. 2D). As presented in Fig. 2E, we measured the expression of angiogenic genes, including *VEGF* and *FGF2*, in L-ADSCs at various time-points post-treatment with 0, 1, 2, and 3 µg/mL of AINs (Fig. 2E). The gene expression levels of *VEGF* and *FGF2* in L-ADSCs were highest at 12 h when the cells were treated with 3 µg/mL of AINs. Apoptosis-related genes, including *BAX* and *CASPASE3*, were significantly upregulated in L-ADSCs that were treated with 5 µg/mL of AINs (Fig. 2F). Additionally, the proliferation-related genes *KI-67* and *PCNA* were significantly downregulated in L-ADSCs that were treated with 5 µg/mL of AINs (Fig. 2G). However, there was no significant difference in the expression levels of both cell apoptosis-related and proliferation-related genes between cells treated with 0 and 3 µg/mL of AINs.

### Functionality-restoring effects of AINs

**Fig. 3:**
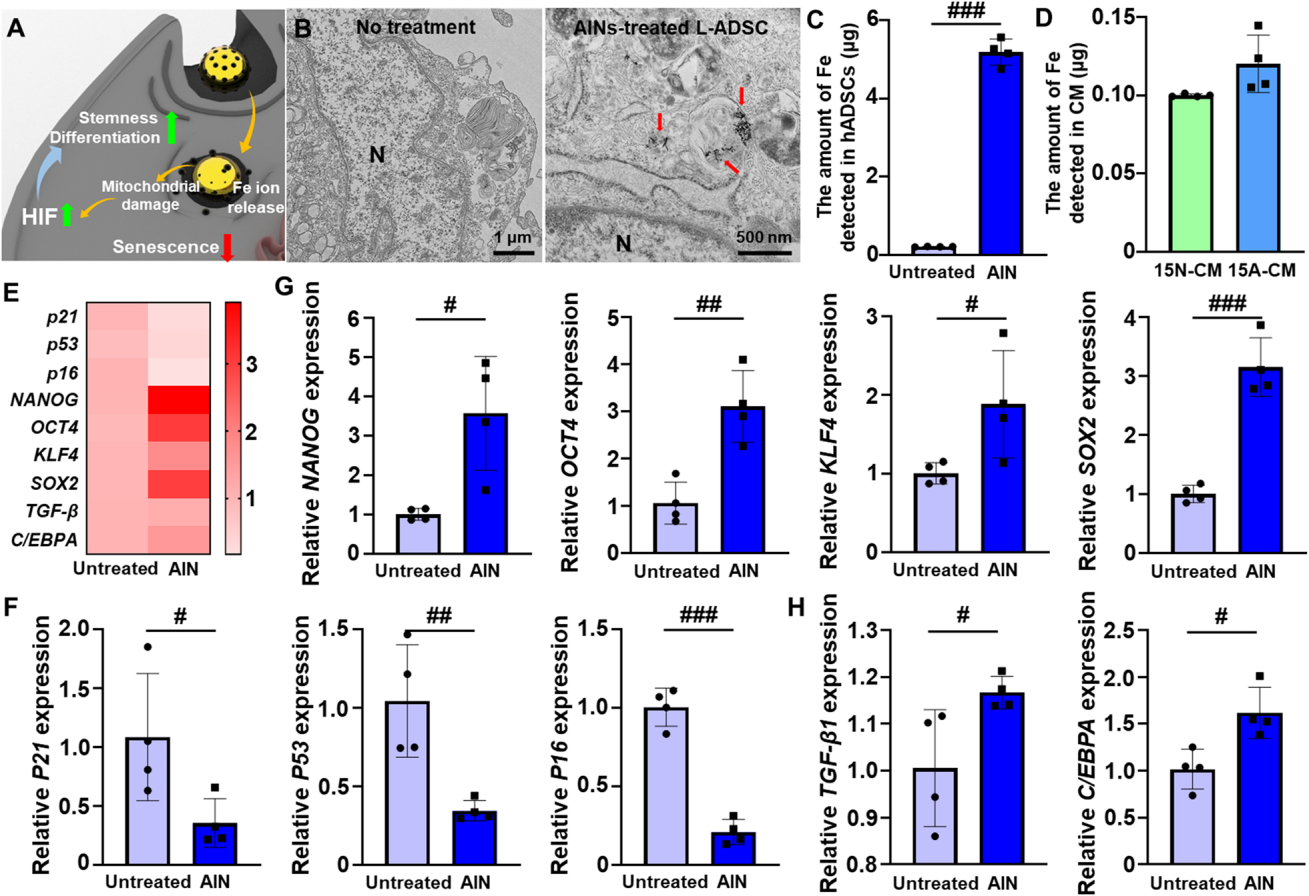
Cellular uptake and effects of AINs in L-ADSCs.** A** Schematic representation of the functionality-restoring effects of AINs. **B** TEM images of L-ADSCs after treatment with AINs for 12 h (N: nucleus, red arrow: intracellular AINs). **C** Mass detection of iron in L-ADSCs with or without AIN treatment as quantified using inductively coupled plasma optical emission spectrometer (ICP-OES; n = 4). **D** Mass detection of iron ions in CM harvested from L-ADSCs at passage 15 without AIN treatment (15 N-CM) and from L-ADSCs at passage 15 with AIN treatment (15 A-CM) as quantified by ICP-OES (n = 4). **E** Heat map image of the expression levels of senescence-related and stemness-related genes. **F** Relative mRNA expression of the senescence-related genes *P21*, *P53*, and *P16* in L-ADSCs with or without AINs treatment (n = 4). **G** Relative mRNA expression of the stemness-related genes *NANOG, OCT4, KLF4*, and *SOX2* in L-ADSCs with or without AIN treatment (n = 4). **H** Relative mRNA expression of chondrogenesis-related and adipogenesis-related genes in L-ADSCs with or without AIN treatment (n = 4). (^#^*p* < 0.05, ^##^p < 0.01, and ^###^*p* < 0.001 versus each group)

The AINs treated the cells can be delivered to intracellular domain through endocytosis. Afterward, the AINs release Fe ions due to low pH condition of endosome. Released Fe ions induce mild ROS generation through affecting mitochondria which results HIF gene expression. The treatments with AINs induced the upregulation of stemness- and differentiation-related gene expression levels along with a downregulation of senescence-relate gene expression in L-ADSCs (Fig. 3A). To investigate the cellular uptake process for AINs, we observed the cytoplasm of L-ADSCs using TEM (Fig. 3B and Additional file 1: Fig. S2). Twelve hours after treatment, AINs were observed within the endosomes of L-ADSCs. To quantify the amount of AINs within the L-ADSCs, the cells were analyzed using an inductively coupled plasma optical emission spectrometer (ICP-OES) (Fig. 3C). Based on this analysis, 5.19 ± 0.33 µg of iron was detected in the L-ADSCs. Additionally, the amounts of iron ions in CM harvested from L-ADSCs at passage 15 without AIN treatment (15 N-CM) and from L-ADSCs at passage 15 with AIN treatment (15 A-CM) were analyzed (Fig. 3D). We observed that the majority of the AINs were retained within L-ADSCs, and based on this observation, we did not focus extensively on the possible iron effect during CM treatment at the wound site. In particular, the heat map image revealed downregulation of senescence-related gene expression and upregulation of stemness- and differentiation-related gene expression in AIN-treated L-ADSCs compared to the expression levels in untreated L-ADSCs (Fig. 3E). AINs treatment significantly decreased the gene expression of senescence-related markers, including *P16*, *P21*, and *P53*, and increased the gene expression of the stemness-related markers *NANOG*, *OCT4*, *KLF4*, and *SOX2* as evaluated using quantitative reverse transcription polymerase chain reaction (qRT-PCR) analyses (Fig. 3F and G). As mentioned earlier, senescence- and stemness-related genes are regulated by HIF. HIF has also been reported to regulate the expression of several differentiation-related genes [42, 43]. The expression levels of differentiation-related genes, including transforming growth factor-beta 1 (TGF-β1) and CCAAT/enhancer-binding protein α (C/EBPA) the function as chondrogenesis and adipogenesis factors, respectively, were increased in L-ADSCs that were treated with AINs compared to the levels in untreated L-ADSCs (Fig. 3 H). Upon treatment, the AINs were successfully delivered to the intracellular domain of L-ADSCs without any detectable extracellular leakage of iron, and these AINs exhibited showed meaningful senescence-overcoming potency by downregulating senescence-related gene expression and upregulating stemness- and differentiation-related gene expression.

### Restoration of angiogenic functionality by AINs

**Fig. 4:**
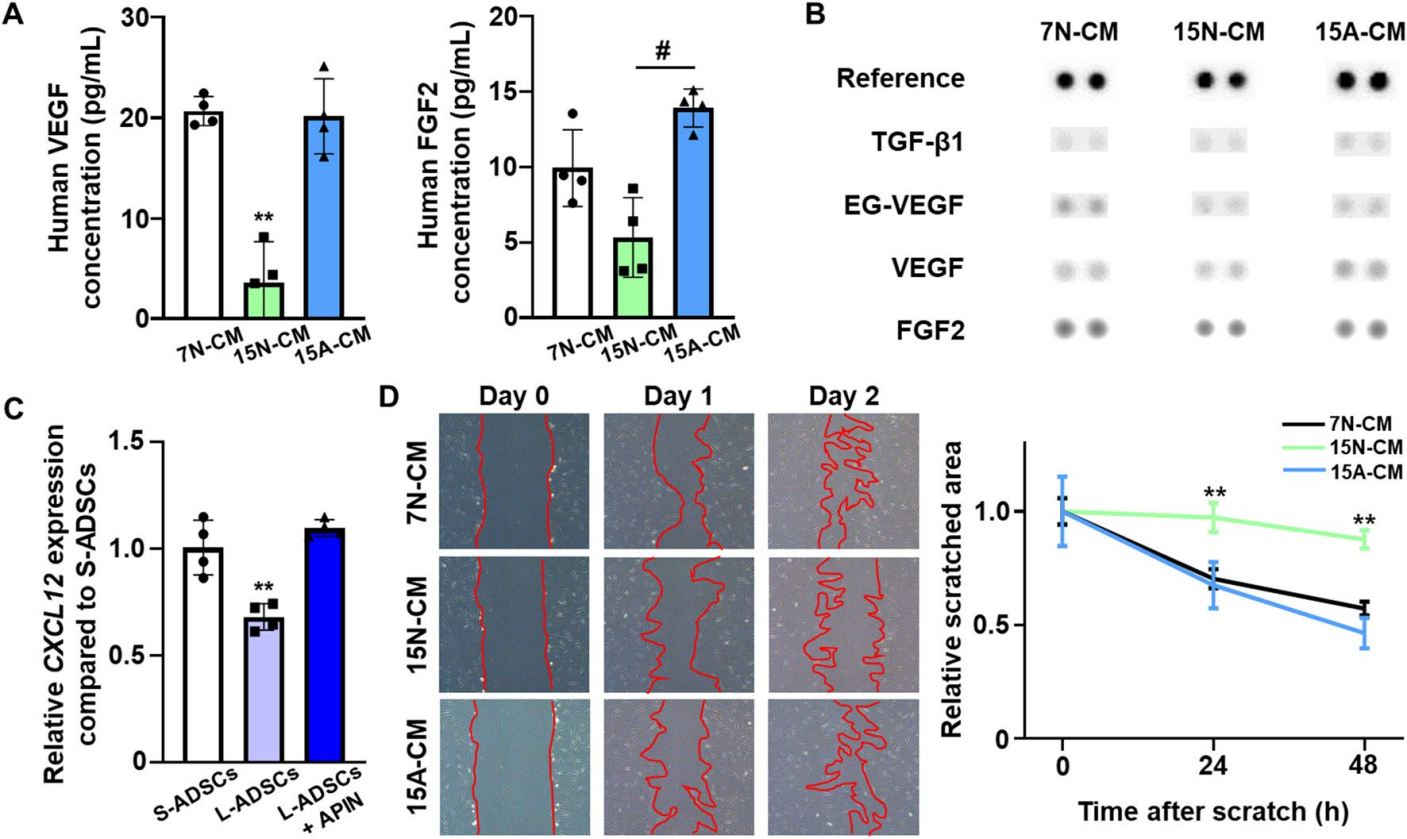
Enhanced angiogenic potency of AINs-treated L-ADSCs.** A** Quantification of human VEGF and FGF2 protein secretion within CM harvested from S-ADSCs at passage 7 without AIN treatment (7 N-CM, 15 N-CM, and 15 A-CM) as evaluated using enzyme-linked immunosorbent assay (ELISA; n = 4, **p < 0.01 versus no treatment group, ^#^*p* < 0.01 versus each group). **B** Representative profiles of angiogenesis-related proteins secreted from 7 N-CM, 15 N-CM, and 15 A-CM as evaluated using a human angiogenesis antibody array. **C** Relative mRNA expression of *CXCL12* in S-ADSCs and in L-ADSCs with or without AINs treatment. as quantified using qRT-PCR after treatment with AINs for 12 h (n = 4, **p < 0.01 versus no treatment group). **D** Representative images of scratched area and relative cell migration area after treatment with 7 N-CM, 15 N-CM, and 15 A-CM as analyzed using a scratch wound assay (red area indicates the scratched area, n = 4, **p < 0.01 versus no treatment group)

To quantify the angiogenic paracrine factor secretion from L-ADSCs post-AINs treatment, VEGF and FGF2, both of which are representative angiogenic factors, were analyzed in CM harvested from S-ADSCs at passage 7 without AIN treatment (7 N-CM, 15 N-CM, and 15 A-CM) using an enzyme-linked immunosorbent assay (ELISA) (Fig. 4A). The concentrations of VEGF and FGF2 in 15 A-CM were significantly higher than those in 15 N-CM, and the concentration in 15 N-CM was similar to that in 7 N-CM. Additionally, the profiles of angiogenesis-related proteins, including TGF-β1, endocrine gland-derived vascular endothelial growth factor (EG-VEGF), VEGF, and FGF2, indicated that the concentrations of these proteins were higher in 15 A-CM than they were in 15 N-CM (Fig. 4B). As presented in Fig. 4C, the gene expression of C-X-C motif chemokine 12 (*CXCL-12*) that is related to cell migration was markedly increased in L-ADSCs that were treated with AINs compared to levels in the untreated L-ADSCs. As senescence is known to decrease cell migration ability [44], we performed scratch wound assays using 7 N-CM, 15 N-CM, and 15 A-CM treatments in hADSCs (Fig. 4D). The 15 A-CM significantly enhanced the migration of hADSCs in a manner that was similar to that of 7 N-CM.

### Enhanced therapeutic ability of 15 A-CM

**Fig. 5:**
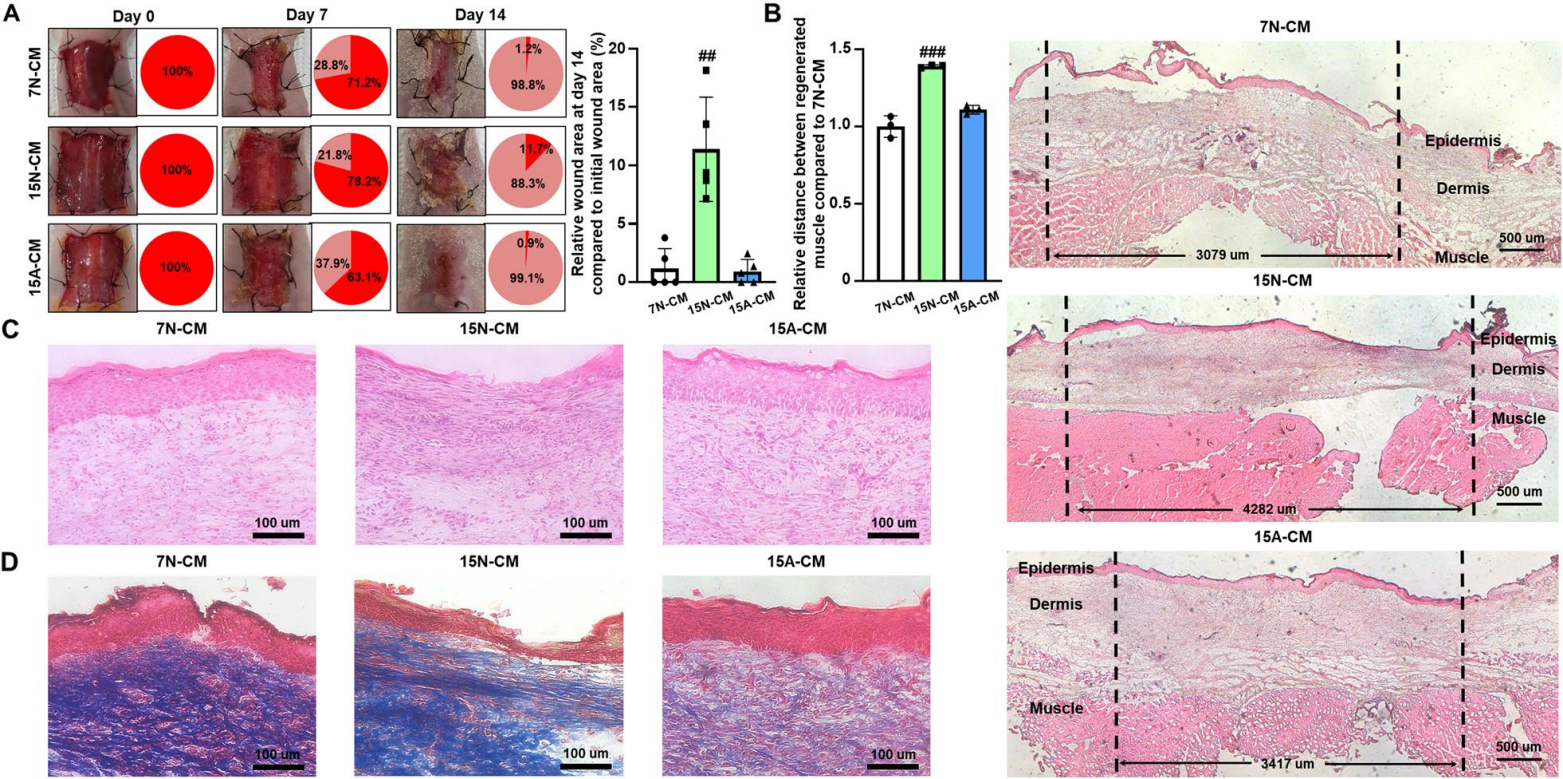
Increased wound-healing effect of 15 A-CM at 14 d after treatment.** A** Images of wounds and wound-closing percentages at 14 d after treatment with CM (n = 5, ^##^p < 0.01 versus other groups). **B** Representative images and quantification of muscle regeneration at 14 d after treatment with CM (n = 3, ^###^p < 0.001 versus other groups). Representative histological images of **C** hematoxylin and eosin (H&E) and **D** Masson’s trichrome (MT) staining of wounds at 14 d after treatment with CM

To verify the therapeutic efficacy of CM, the closed wound sizes, degrees of regenerated muscle, and complete formation of dermis and epidermis were evaluated at 14 d after surgery. Each CM was injected at a volume of 200 µL daily for the initial 4 d period. At 11 d after the last CM injection, the wounds of the 7 N-CM- and 15 A-CM-injected groups were healed by approximately 99% compared to the size initial wound area, while the wounds of the 15 N-CM-injected group were healed by approximately 88% (Fig. 5A). The degree of regenerated muscle was evaluated by measuring the distance between the edges of the defect. The distance between regenerated muscles for the 15 A-CM-injected group was approximately 3,417 μm on an average, and this was significantly shorter compared to that of the 15 N-CM-injected group (Fig. 5B). Hematoxylin and eosin (H&E) staining and Masson’s trichrome (MT) staining of skin tissue at day 14 revealed complete formation of dermis and epidermis with reduced fibrosis and inflammation in the 15 A-CM-injected group (Fig. 5C and D). The 15 N-CM-injected group clearly exhibited less of dermis and epidermis formation and a greater degree of fibrosis and inflammation compared to these values in the 7 N-CM- and 15 A-CM-injected groups.

**Fig. 6:**
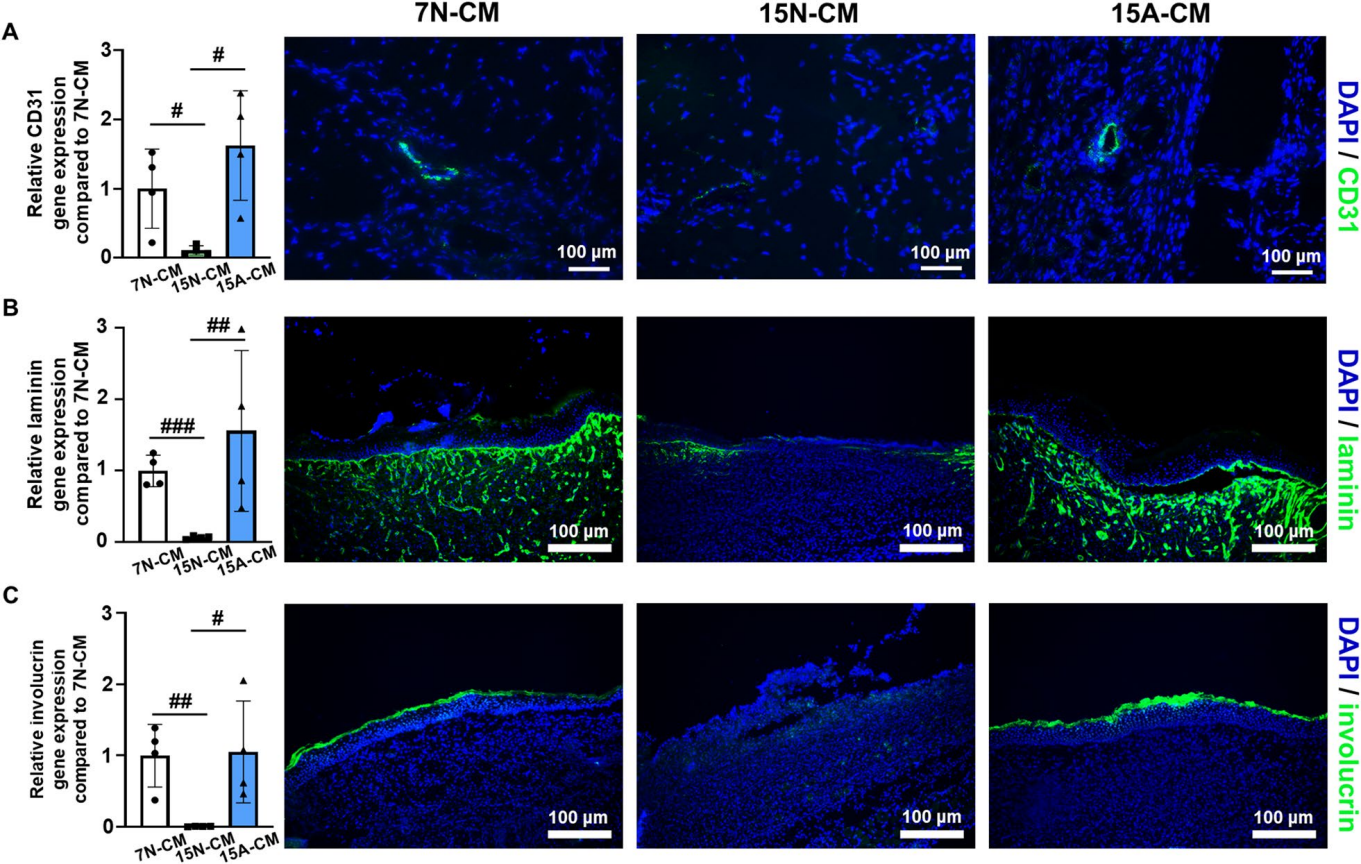
Improved angiogenesis and dermal regeneration at 14 d after 15 A-CM treatment within the wound tissue.** A** Analysis of a vascular marker (CD31) using qRT-PCR and immunostaining (n = 3, blue: nucleus, green: CD31). **B** Analysis of a representative dermis marker (laminin) using qRT-PCR and immunostaining (n = 3, blue: nucleus, green: laminin). **C** Analysis of a representative epidermis marker (involucrin) using qRT-PCR and immunostaining (n = 3, blue: nucleus, green: involurin) (^#^*p* < 0.05, ^##^*p* < 0.01, and ^###^*p* < 0.001 versus each group)

To determine the extent of angiogenesis and dermal regeneration within the wound tissues, blood vessels and dermal formation-related markers in the defect areas were analyzed using qRT-PCR and immunostaining. The 15 A-CM-injected group expressed *CD31* to a greater degree and possessed more distinct microvessels that were immunostained with anti-CD31 antibodies compared to these values in the 15 N-CM-injected group (Fig. 6A). Similar to the observations regarding CD31, the 15 A-CM-injected group exhibited significantly higher gene expression and more obvious detection of laminin and involucrin (major protein components of the dermis and substrate protein, respectively) within the keratinocytes of the epidermis (Fig. 6B and C).

## Conclusions

In the present study, we designed a novel anti-senescence nanomaterial that can respond to low pH conditions and release iron ions in an intracellular manner to recover the therapeutic properties of L-ADSCs. Iron ions released from AINs in response to the low pH condition present within the endosomes induce mild ROS generation in L-ADSCs and upregulate HIF-related pathways that can enhance angiogenic paracrine factor secretion. Interestingly, L-ADSCs treated with AINs exhibited downregulated senescence-related gene expression and upregulated stemness- and differentiation-related gene expression without the occurrence of extracellular iron leakage. The secretion of angiogenic paracrine factors by L-ADSCs treated with AINs was significantly enhanced to levels that were similar to those observed in S-ADSCs. As a result of the enhanced secretion of angiogenic paracrine factors, CM harvested from L-ADSCs that were treated with AINs displayed drastically enhanced therapeutic results compared to those observed in response to CM harvested from conventional L-ADSCs. Consequently, L-ADSCs that were previously considered to be of no use can now be utilized based on treatment of these cells with AINs. Thus, the AINs described in this study may serve as a safe, economical, and progressive strategy for promoting stem cell-based therapy and senescence studies.

## Methods

### Materials

All materials used for the synthesis of AIN, including polyvinylpyrrolidone (PVP), NaBH_4_, HAuCl_4_∙xH_2_O (99.995%), and FeCl_3_ (98%), were purchased from Sigma-Aldrich (St. Louis, MO, USA).

### Synthesis of AINs

To synthesize AINs, a PVP solution (9 mL of deionized [DI] water + 100 mg of PVP) was prepared one day prior to synthesis under magnetic stirring (600 rpm) at approximately 25 °C. Prior to synthesis, 4 mg of NaBH_4_, 2 mg of FeCl_3_ and 4 mg of HAuCl_4_∙xH_2_O, were dissolved in 1 mL of DI water. Subsequently, 1 mL of NaBH_4_ solution was added to 9 mL of the previously prepared PVP solution and stirred at 600 rpm and at approximately 25 °C for 10 min. Thereafter, the prepared FeCl_3_ (1 mL) and HAuCl_4_∙xH_2_O solutions (1 mL) were injected drop-wise using a micro pipette into the reaction solution. The solution (12 mL) was allowed to react under magnetic stirring (600 rpm) at approximately 25 °C for an additional 15 min. The reacted solution was subjected to centrifugation (8000 rpm, 10 min) to precipitate the AINs, and then washed alternately with DI water and acetone twice. The final AINs were dispersed into 50 mL of DI water.

### Characterization

An emission electron microscope (JEM-2100 F, JEOL, Tokyo, Japan) was used to obtain TEM and EDXS images (200 kV). The TEM images were analyzed to determine the size distribution of the AINs. An X-ray diffractometer (D-MAX/A, Rigaku, Tokyo, Japan) was used to capture powder XRD patterns at 35 kV and 35 mA. The UV-Vis spectrum in the range of 250–850 nm was analyzed in a crystal cube using a UV–Vis spectrophotometer (Cary 60 UV–Vis, Agilent Technologies, Santa Clara, CA, USA). The elemental amounts of gold and iron were directly measured using an inductively coupled plasma (ICP) spectrometer (Direct Reading Echelle ICP, Teledyne Leeman Labs, Hudson, NH, USA).

### Fe ion leakage from acid-treated AINs used to mimic endosome conditions

A pH of 4.5 was selected to assess Fe ion release from AINs [19]. The AINs were exposed to different pH conditions (pH 4.5 and 7.0) and dispersed in phosphate-buffered saline solution (PBS; Gibco BRL, Gaithersburg, MD, USA) that was adjusted using hydrochloric acid (HCl 37%, Sigma-Aldrich). The AINs were exposed for 12 h, as this time point was similar to that used for the angiogenic gene expression data. The remaining AINs in PBS were extracted by centrifugation (8000 rpm, 10 min). The amount of Fe ions that was released from the AINs was determined using a UV-Vis spectrophotometer and an ICP spectrometer by analyzing the altered Au/Fe ratio in the AINs.

### hADSC culture

hADSCs were purchased from Lonza (Bazel, Switzerland) and cultured in Dulbecco’s Modified Eagle’s Medium (DMEM; Gibco BRL) supplemented with 10% (v/v) fetal bovine serum (Gibco BRL), and 1% (v/v) penicillin/streptomycin (Gibco BRL). The cells were cultured at 37 °C in a humidified incubator with 5% (v/v) CO_2_. The medium was changed every 2 d. To treat the hADSCs with AINs, a serum-free medium was used to eliminate potential disturbance in the surface charges of the AINs that can be affected by the proteins in the serum [45].

### Cell viability

Cell viability was determined using the CCK-8 assay (Dojindo Molecular Technologies Inc., Rockville, MD, USA) that measures the amount of formazan dye that is reduced by the intracellular dehydrogenase activity. The number of living cells is proportional to the amount of formazan dye. Briefly, h-ADSCs (1 × 10^4^ cells/well) were cultured for 24 h in 24-well plates with various concentrations of AINs, and this was followed by three consecutive washes using PBS. The CCK-8 solution was mixed with fresh serum-free medium and added into each well. After 2 h of incubation, the samples were measured using a plate reader (Infinite F50, Tecan, Zurich, Switzerland) at an absorbance of 450 nm. Cell viability was calculated as the percentage of the viable cells in the groups treated with AINs relative to that in the untreated groups (*n* = 4 per group).

### Cell morphology

Cell morphology was evaluated using 1,1′-dioctadecyl-3,3,3′,3′-tetramethylindocarbocyanine perchlorate (DiI; Thermo Fisher Scientific, Waltham, MA, USA) staining. After treating the cells with various concentrations of AINs for 24 h, the cells were incubated for 2 h at 37 °C with DiI (6.25 µM) and then washed twice with PBS. The cells were fixed with 4% paraformaldehyde solution for 10 min and washed again with PBS. After 4,6-diamidino-2-phenylindole (DAPI; Thermo Fisher Scientific) staining, DiI fluorescence was measured using a fluorescence microscope (DMi8, Leica, Wetzlar, Germany).

### Terminal deoxynucleotidyl transferase dUTP nick end labeling assay

To observe the apoptotic activity of L-ADSCs that were treated with AINs for 24 h, the Fluorescein in situ Apoptosis Detection Kit (Merck Millipore, Darmstadt, Germany) was used according to the manufacturer’s instructions. The final fluorescence images were captured using a fluorescence microscope (DMi8).

### Harvest of CM

hADSCs (1.6 × 10^6^ cells) were seeded into a 150 mm cell culture plate (Corning Inc., Corning, New York, NY, USA) and incubated overnight. The medium was replaced with medium containing AINs after incubation for 24 h, and the cells were then incubated for an additional 12 h. After incubation, the cells were washed three times with PBS and cultured for 2 d in serum-free DMEM. To remove any remaining cell fragments and contaminants, the harvested CM was subjected to centrifugation (1500 rpm, 5 min) and filtration.

### Cellular uptake and leakage of AINs

Cellular uptake and leakage of AINs in L-ADSCs (1.0 × 10^5^ cells) were analyzed by quantifying the amount of gold and iron ions within the cells and CM. After uptake of AINs (12 h), the cells were washed and lysed in nitric acid hydrochloride (a mixture of nitric acid and hydrochloric acid in a molar ratio of 1:3) to dissolve all of the components, including AINs. Ionized samples were diluted in DI water (1:4 [v/v]). The gold and iron concentrations were determined using an ICP-OES (Varian, Palo Alto, CA, USA). To observe the uptake of AINs, L-ADSCs were seeded into a 6-well plate and incubated with AINs. Ultrathin sections of the cells were analyzed using TEM (Talos L120C, Thermo Fisher Scientific) at 120 kV to observe the distribution of AINs. Briefly, AIN-treated cells were washed three times with PBS to eliminate the unbound AINs. The cells were treated with trypsin, washed again three times with PBS, and fixed with Karnovsky’s fixative (5% glutaraldehyde [Sigma-Aldrich] + 4% formaldehyde in 0.1 M cacodylate buffer [Sigma-Aldrich] + 50 mg CaCl_2_ [Sigma-Aldrich]/100 mL H_2_O) for 2 h. The fixed cells were washed three times with 0.05 M sodium cacodylate buffer. Post-fixation staining was performed using 2% osmium tetroxide (Sigma-Aldrich) in 0.1 M cacodylate buffer for 2 h at 4 °C. The samples were dehydrated in alcohol (30%, 50%, 70%, 80%, 90%, and 100% ethanol), treated twice with propylene oxide (Sigma-Aldrich) for 10 min, and then treated with propylene oxide and Spurr low-viscosity resin for 2 h. The samples were further treated for 24 h with pure resin and then embedded in molds. The resin blocks were polymerized at 70 °C for 2 d and then cut into ultrathin sections (70 nm) using an ultramicrotome (Reichert-Jung Ultracut E, Leica). The sections were stained with 1% lead citrate and 0.5% uranyl acetate and then analyzed. Cellular uptake time was determined based on the gene expression results.

### Quantitative reverse transcription polymerase chain reaction

qRT-PCR was used to quantify the relative expression levels of genes encoding glyceraldehyde 3-phosphate dehydrogenase (*GAPDH*), *VEGF*, *FGF2*, Bcl-2-associated X protein (*BAX*), *caspase3*, *ki-67*, proliferating cell nuclear antigen (*PCNA*), *P21*, *P53*, *P16*, *NANOG*, octamer-binding transcription factor 4 (*OCT4*), kruppel-like factor 4 (*KLF4*), *SOX2*, C-X-C chemokine receptor type 4 (*CXCR4*), β-actin, cluster of differentiation 31 (*CD31*), *laminin*, and *involucrin*. The samples were lysed in TRIzol™ reagent (Invitrogen, Carlsbad, CA, USA), and total RNA was extracted with chloroform and precipitated with isopropanol. After the supernatant was removed, the RNA pellet was washed with 75% (v/v) ethanol, air-dried, and dissolved in 0.1% (v/v) diethyl pyrocarbonate (DEPC)-treated water. For qRT-PCR, the SsoAdvanced™ Universal SYBR Green Supermix Kit (Bio-Rad, Hercules, CA, USA) and the CFX Connect™ real-time PCR Detection System (Bio-Rad) were used. The primers used for qRT-PCR are listed in Table 1.

**Table 1:** Sequences of qRT-PCR primers

Primer	Sequence	
Human *GAPDH*	Forward	5′-GTC GGA GTC AAC GGA TTT GG-3′
	Reverse	5′-GGG TGG AAT CAA TTG GAA CAT-3′
Human *VEGF*	Forward	5′-GAG GGC AGA ATC ATC ACG AAG T-3′
	Reverse	5′-CAC CAG GGT CTC GAT TGG AT-3′
Human *FGF2*	Forward	5′-GAC GGC AGA GTT GAC GG-3′
	Reverse	5′-CTC TCT CTT CTG CTT GAA GTT-3′
Human *BAX*	Forward	5′-GCA ACT TCA ACT GGG GCC GGG-3′
	Reverse	5′- GAT CCA GCC CAA CAG CCG CTC-3′
Human *CASPASE3*	Forward	5′-CCT GGT TAT TAT TCT TGG CGA AA-3′
	Reverse	5′-GCA CAA AGC GAC TGG ATG AA-3′
Human *KI-67*	Forward	5′-CCA CAC TGT GTC GTC GTT TG-3′
	Reverse	5′-CCG TGC GCT TAT CCA TTC A-3′
Human *PCNA*	Forward	5′-CCT GCT GGG ATA TTA GCT CCA-3′
	Reverse	5′-CAG CGG TAG GTG TCG AAG C-3′
Human *P21*	Forward	5’-TGA GCC GCG ACT GTG ATG-3’
	Reverse	5′-GTC TCG GTG ACA AAG TCG AAG TT-3′
Human *P53*	Forward	5′-CCT CAG CAT CTT ATC CGA GTG G-3′
	Reverse	5′-TGG ATG GTG GTA CAG TCA GAG C-3′
Human *P16*	Forward	5′-ACT TCA GGG GTG CCA CAT TC-3′
	Reverse	5′-CGA CCC TGT CCC TCA AAT CC-3′
Human *NANOG*	Forward	5′-AGT CCC AAA GGC AAA CAA CCC ACT TC-3′
	Reverse	5′-TGC TGG AGG CTG AGG TAT TTC TGT CTC-3′
Human *OCT4*	Forward	5′-CTG GGT TGA TCC TCG GAC CT-3′
	Reverse	5′-CAC AGA ACT CAT ACG GCG GG -3′
Human *KLF4*	Forward	5′-TCT CAA GGC AGA CCT GCG AA-3′
	Reverse	5′-TAG TGC CTG GTC AGT TCA TC-3′
Human *SOX2*	Forward	5′-TGA TGG AGA CGG AGC TGA A-3′
	Reverse	5′-GGG CTG TTT TTC TGG TTG C-3′
Human *TGF-β1*	Forward	5′-CCC AGC ATC TGC AAA GCT C-3′
	Reverse	5′-GTC AAT GTA CAG CTG CCG CA-3′
Human *C/EBPA*	Forward	5′-TTG TGC CTT GGA AAT GCA AAC-3′
	Reverse	5′-TCG GGA AGG AGG CAG GAA AC-3′
Human *CXCR12*	Forward	5′-TGC CAG AGC CAA CGT CAA G-3′
	Reverse	5′-CAG CCG GGC TAC AAT CTG AA-3′
Mouse *β-actin*	Forward	5′-GGC TGT ATT CCC CTC CAT CG-3′
	Reverse	5′-CCA GTT GGT AAC AAT GCC ATG T-3′
Mouse *CD31*	Forward	5′-CAA ACA GAA ACC CGT GGA GAT G-3′
	Reverse	5′-ACC GTA ATG GCT GTT GGC TTC-3′
Mouse *Laminin*	Forward	5′-GGA CGG GAA TTC CGT TAG GG-3′
	Reverse	5′-CAG GTC CAA GGA CTG CAC TT-3′
Mouse *Involucrin*	Forward	5′-CCT GTG AGT TTG TTT GGT CTA CA-3′
	Reverse	5′-GAA CCA CAG CTG GAA CAG TC-3′

### CM component analysis

The protein components of 7 N-CM, 15 N-CM, and 15 A-CM were identified using a man angiogenesis array kit (R&D Systems, Inc., Minneapolis, MN, USA). One mL of CM was loaded onto the human angiogenesis array kit. After blocking the array membrane with blocking buffer for 1 h and subsequent membrane washing, each CM and array detection antibody cocktail was mixed and added to the blocked membrane. This was followed by overnight incubation at 4 °C with shaking. After washing, streptavidin-horseradish peroxidase (HRP) buffer was added to the membrane, and the membrane was incubated for 30 min. Following another wash, Chemi Reagent Mixture was added to the membrane for reaction at room temperature, and the signal was measured using the LAS-3000 system (Fujifilm, Tokyo, Japan).

### Cell migration

To evaluate the migration ability of each CM, hADSCs (1 × 10^5^ cells) were seeded into 6-well cell culture dishes (Corning Inc.). The cells were incubated for 24 h and then scratched. The migration area was measured at 0, 12, and 24 h after scratching. The relative migration area was calculated based on comparison to the initial scratch areas.

### Mouse skin wound model

Four-week-old female athymic mice (BALB/c-nu, 20–25 g body weight; Orient, Seongnam, Gyeonggi, Korea) were anesthetized with xylazine (10 mg/kg, Bayer, Seoul, Korea) and ketamine (100 mg/kg, Yuhan, Seoul, Korea). Each group included six, randomly selected mice. The full-thickness skin was excised in a square shape (2 × 2 cm) and ligated using a 6-0 silk suture (AILEE, Busan, Korea) at two sites on each border. CM (200 µL) was injected into the muscles on each side using the same volume every day for the initial 4 d period. Based on this, 800 µL of CM was injected into each mouse. The wound was covered with sterile gauze after the CM injections. The wound-covering films were changed at 3, 7, and 10 d to acquire photographs of the wounds. All animals were sacrificed at 14 d after anesthesia to harvest the wound tissue. All animal treatments and experimental procedures were approved by the Institutional Animal Care and Use Committee of Sungkyunkwan University (no. SKKUIACUC2017-05-03).

### Histological examination

The wound tissue that was retrieved at 14 d post-surgery was embedded in optimal cutting temperature compound (SciGen Scientific Inc., Gardenas, California, USA). The samples were frozen at − 20 °C and sliced into 10 μm-thick sections using a microtome (Cryostat, Leica). The sliced samples were stained with H&E to verify tissue regeneration and with MT staining to assess inflammation.

### Immunohistochemistry

The sliced samples were stained with primary antibodies targeting anti-CD31, anti-laminin, and anti-involucrin antibodies (Abcam, Cambridge, UK). After staining with the primary antibodies, fluorescein isothiocyanate-conjugated anti-rabbit antibodies (Jackson ImmunoResearch Laboratories, West Grove, PA, USA) were used to detect the signals. The immunohistochemically stained samples were counterstained with DAPI, and the images were obtained using a fluorescence microscope (DMi8).

### Statistical analyses

All quantitative data are expressed as mean ± standard deviation. For statistical analysis, one-way analysis of variance was performed with the Bonferroni correction using SigmaPlot (version 12.5, Systat software, San Jose, CA, USA). Statistical significance was set at *p* < 0.05.

## Supplementary Information

**Additional file 1.** TEM images of hADSCs after AINs treatment for 12 h shown as stepwise magnification (N: nucleus, red arrows indicate AINs in a cell).

## Abbreviations

AIN: Anti-senescence ion-delivering nanocarrier
AuNP: Gold nanoparticle
CCK-8: Cell Counting Kit-8
CM: Conditioned media
EDXS: Energy-dispersive X-ray spectroscopy
ELISA: Enzyme-linked immunosorbent assay
FDA/EB: Fluorescein diacetate/ethidium
FGF: Fibroblast growth factor
hADSC: Human adipose-derived stem cell
HIF: Hypoxia-inducible factor
H&E: Hematoxylin and eosin
ICP-OES: Inductively coupled plasma optical emission spectrometer
L-ADSCs: Long-term-cultured hADSCs
MT: Masson’s trichrome
ROS: Reactive oxygen species
S-ADSCs: Short-term-cultured hADSCs
TEM: Transmission electron microscopy
TUNEL: Terminal deoxynucleotidyl transferase-mediated dUTP nick end labeling
UV–Vis: Ultraviolet–visible spectroscopy
VEGF: Vascular endothelial growth factor
XRD: X-ray diffraction
