# Supplementary material for: Anti-senescence ion-delivering nanocarrier for recovering therapeutic properties of long-term-cultured human adipose-derived stem cells
Source: J Nanobiotechnology. 2021 Oct 30;19:352. doi: 10.1186/s12951-021-01098-7 (PMC8557526; doi:10.1186/s12951-021-01098-7)
Supplement: Supplementary file 1 — Additional file 1. TEM images of hADSCs after AINs treatment for 12 h shown as stepwise magnification (N: nucleus, red arrows indicate AINs in a cell). [file 12951_2021_1098_MOESM1_ESM.docx]

**(Supporting Information) Anti-Senescence Ion-Delivering Nanocarrier for Recovering Therapeutic Properties of Long-Term-Cultured Human Adipose-Derived Stem Cells**

Yeong Hwan Kim^a,†^, Gwang-Bum Im^a,†^, Sung-Won Kim^a^, Yu-Jin Kim^a^, Taekyung Yu^b^, Ju-Ro Lee^c^, Soong Ho Um^a^, Yoon Ki Joung^c,d^, and Suk Ho Bhang^a,*^

*^a^School of Chemical Engineering, Sungkyunkwan University, Suwon 440-746, Republic of Korea*

*^b^Department of Chemical Engineering, College of Engineering, Kyung Hee University, Yongin, 17104, Republic of Korea*

*^c^Center for Biomaterials, Biomedical Research Institute, Korea Institute of Science and Technology, Hwarang-ro 14-gil 5, Seoungbuk-gu, Seoul 02792, Republic of Korea*

*^d^Division of Bio-Medical Science & Technology, University of Science and Technology, 113 Gwahangno, Yuseong-gu, Daejeon 305-333, Republic of Korea*

† **Co-first authors:** These authors contributed equally to this work.

***Corresponding author**

Suk Ho Bhang, Ph.D.

E-mail: sukhobhang@skku.edu; Tel.: +82-31-290-7242, Fax: +82-31-290-7272

**
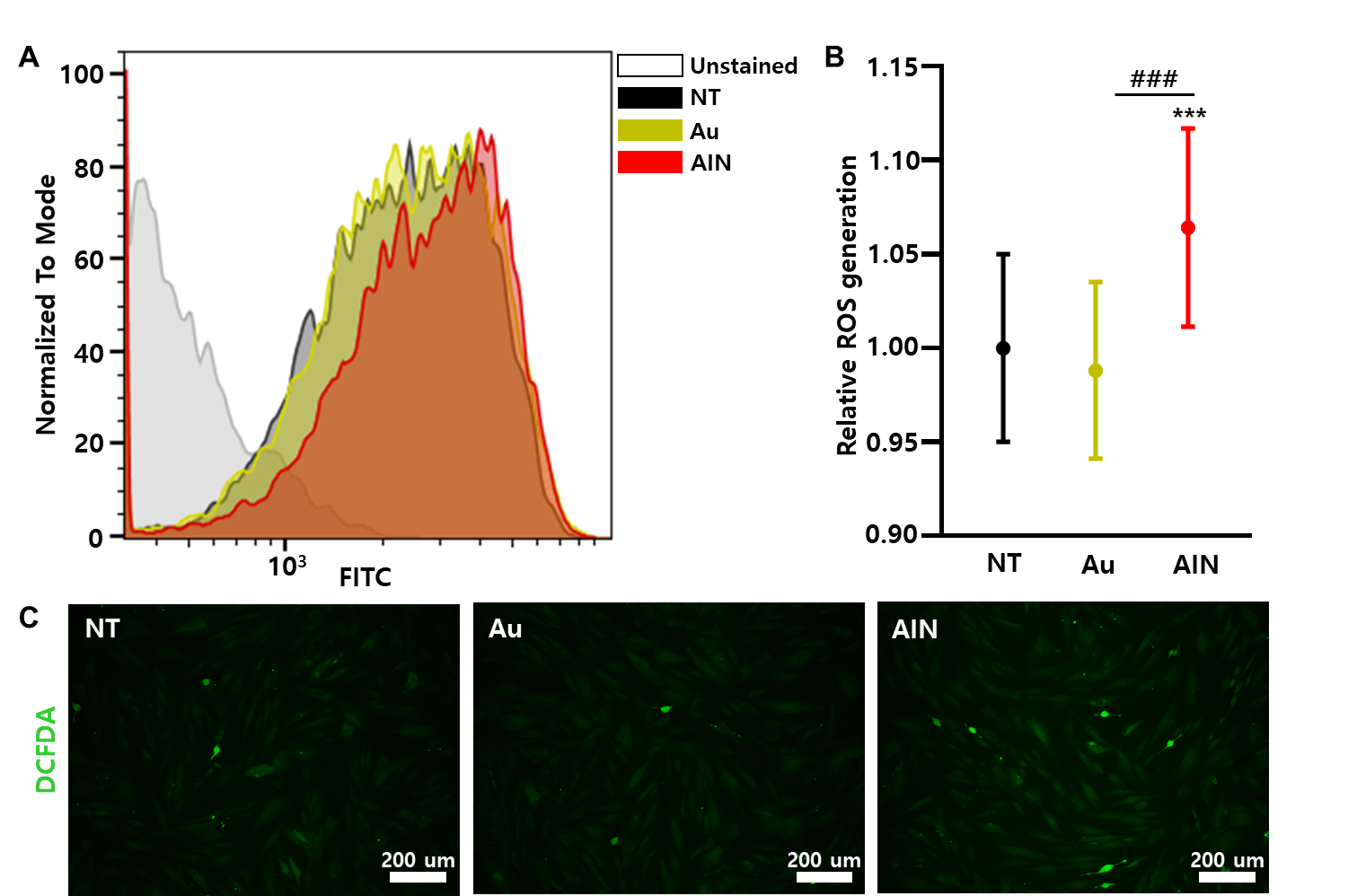
**

**Supplementary figure 1. Cellular reactive oxygen species (ROS) generation of anti-senescence ion-delivering nanocarriers** **(AINs).** **(A)** Fluorescence-activated cell sorting (FACS) results of human adipose-derived stem cells (hADSCs) with or without the nanoparticle treatments. The cells were stained with fluorescein isothiocyanate (FITC)-conjugated dichlorofluorescein diacetate (DCFDA). **(B)** Luminescence assay of ROS generation of hADSCs with or without the nanoparticle treatments (n = 85, ****p* < 0.001 versus no treatment group, ###*p* < 0.001 versus each group). **(C)** Cellular ROS staining of hADSCs with or without the nanoparticles treatments (ROS; DCFDA, green).


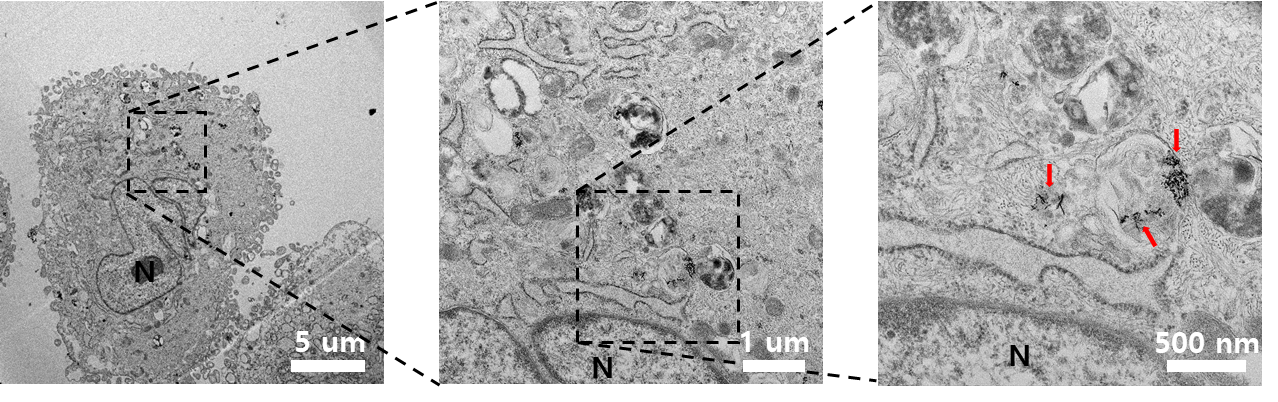


**Supplementary figure 2. Transmission electron microscopy (TEM) images of hADSC treated with AINs** (N: nucleus, red arrows indicate AINs in a cell).


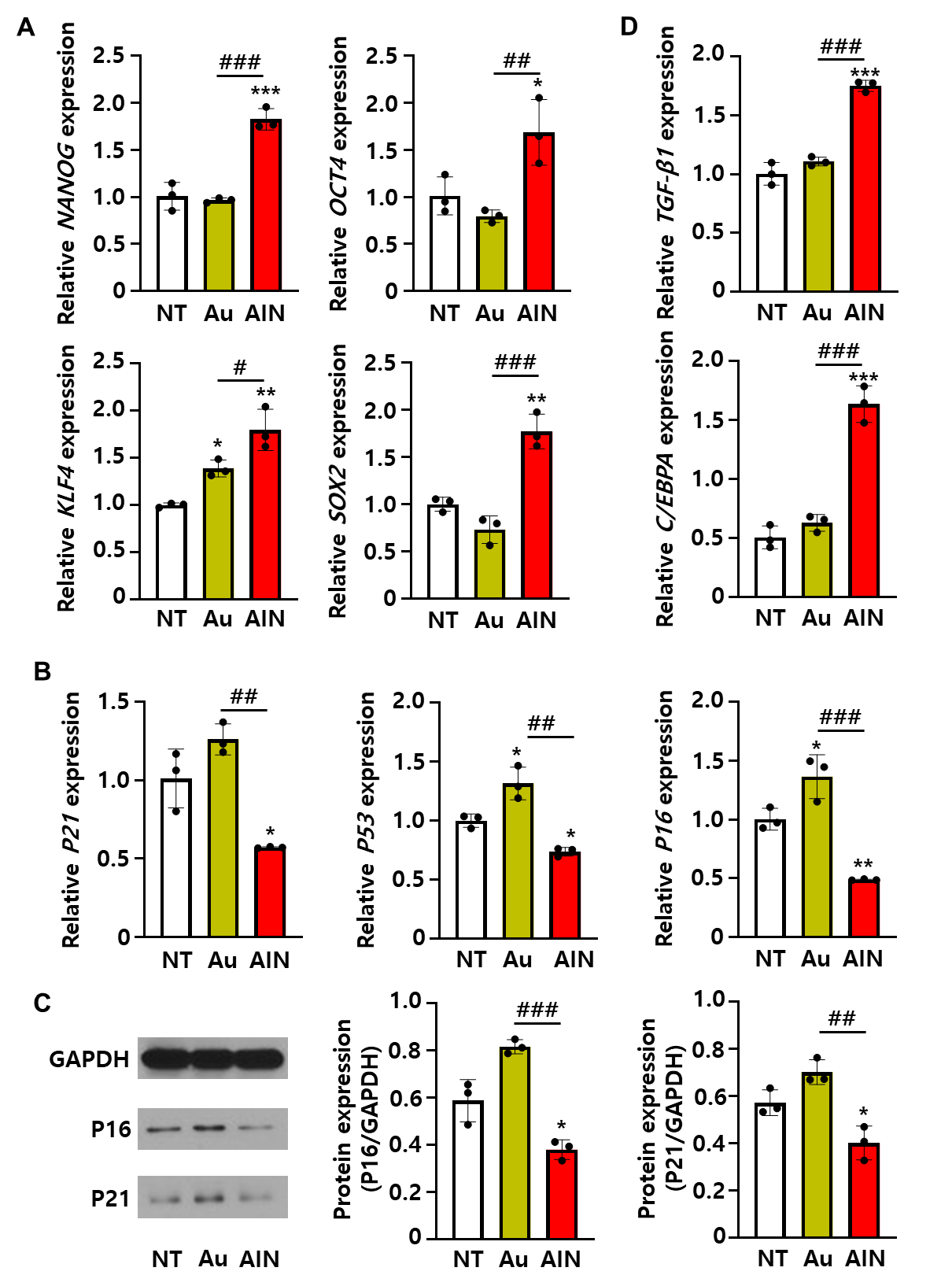


**Supplementary figure 3. Functionality-restoring effect of AIN in L-ADSCs.** **(A)** Relative mRNA expression of the stemness-related genes *NANOG, OCT4, KLF4*, and *SOX2* in L-ADSCs with or without the nanoparticle treatments (n = 3). **(B)** Relative mRNA expression of the senescence-related genes *P21*, *P53*, and *P16* in L-ADSCs with or without the nanoparticle treatments (n = 3). **(C)** Protein expression of the senescence-related proteins P16 and P21 in L-ADSCs with or without of the nanoparticle treatments (n = 3). **(D)** Relative mRNA expression of chondrogenesis- and adipogenesis-related genes in L-ADSCs with or without the nanoparticle treatments (n = 3). **p* < 0.05, ***p* < 0.01, and ****p* < 0.001 versus no treatment, #*p* < 0.05, ##p < 0.01, and ###*p* < 0.001 versus each group.


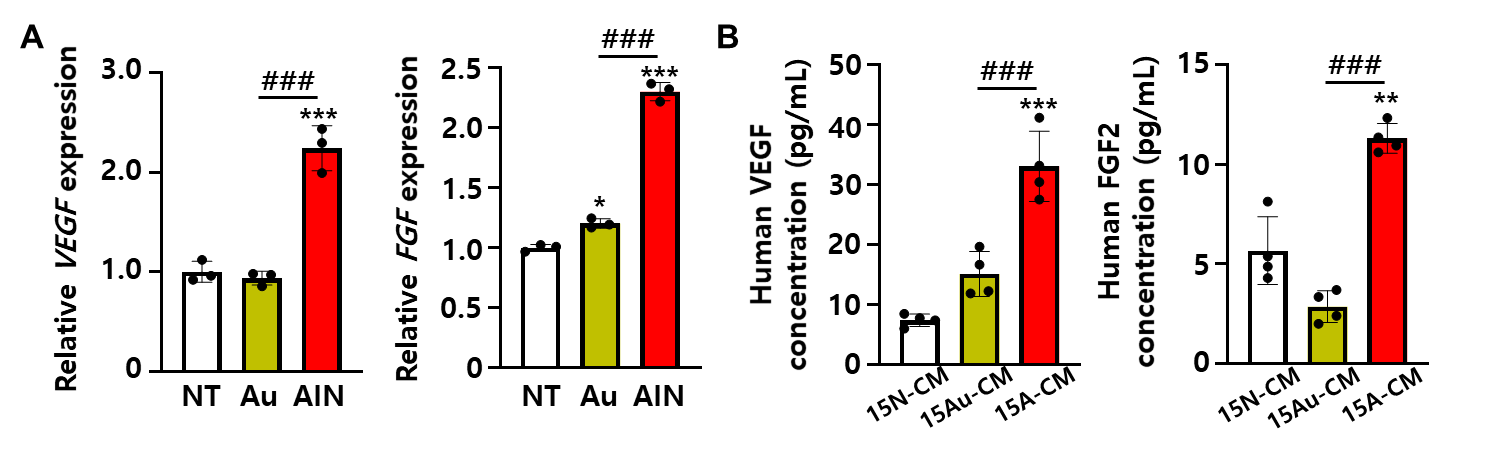


**Supplementary figure 4. Angiogenic effect of iron ions on L-ADSCs.** **(A)** Relative mRNA expression of the stemness-related genes, *VEGF* and *FGF* in L-ADSCs with or without the nanoparticle treatments (n = 3). **(B)** Quantification of human VEGF and FGF2 protein secretion within CM harvested from L-ADSCs with or without the nanoparticle treatments (n = 4). **p* < 0.05, ***p* < 0.01, and ****p* < 0.001 versus no treatment, ###*p* < 0.001 versus each group.

**Methods**

***Reactive oxygen species (ROS) assay***

ROS was measured using the fluorescent indicator DCFDA (D339 Invitrogen, Carlsbad, USA). After AIN treatment, the cells were incubated with 10 μM DCFDA in PBS solution for 20 min at 37 °C. The samples were washed twice with PBS or FACS buffer solution and examined by fluorescence microscopy (DMi8) and FACS using a flow cytometer (MACSQuant® VYB, Miltenyi Biotec, Bergisch-Gladbach, Germany).

***Western blotting***

L-ADSCs were collected and lysed in radioimmunoprecipitation (RIPA) lysis buffer (Rockland Immunochemicals Inc., Limerick, PA, USA) and then centrifuged at 10,000 × g for 10 min; the supernatant was then used as the protein extract for all of the western blotting evaluations. Protein concentrations were determined using a bicinchoninic acid (BCA) protein assay (Pierce Biotechnology, Rockford, IL, USA). An equal amount of protein from each sample was mixed with sample buffer, loaded, and subjected to sodium dodecyl sulfate polyacrylamide gel electrophoresis (SDS-PAGE) using a 10% (w/v in water) resolving gel. Proteins separated by SDS-PAGE were then transferred to a polyvinylidene fluoride membrane (Bio-Rad) and probed with antibodies against GAPDH, P16, and P21 (Abcam) overnight at 4 °C. The membranes were then washed and incubated with a horseradish peroxidase-conjugated secondary antibody (R&D Systems) for 1 h at room temperature. The blots were developed in a darkroom, and luminescence was recorded using X-ray film blue (Agfa HealthCare NV, Mortsel, Belgium). Bands were imaged using Photoshop CC (Adobe Systems).
